# The role of metal accessibility on carbon dioxide electroreduction in atomically precise nanoclusters†

**DOI:** 10.1039/d3sc04085b

**Published:** 2023-10-24

**Authors:** Yingwei Li, Grant J. Stec, Agnes E. Thorarinsdottir, Ryan D. McGillicuddy, Shao-Liang Zheng, Jarad A. Mason

**Affiliations:** a Department of Chemistry & Chemical Biology, Harvard University 12 Oxford Street Cambridge Massachusetts 02138 USA mason@chemistry.harvard.edu

## Abstract

Atomically precise nanoclusters (NCs) can be designed with high faradaic efficiency for the electrochemical reduction of CO_2_ to CO (FE_CO_) and provide useful model systems for studying the metal-catalysed CO_2_ reduction reaction (CO_2_RR). While size-dependent trends are commonly evoked, the effect of NC size on catalytic activity is often convoluted by other factors such as changes to surface structure, ligand density, and electronic structure, which makes it challenging to establish rigorous structure–property relationships. Herein, we report a detailed investigation of a series of NCs [Au_*n*_Ag_46−*n*_(C≡CR)_24_Cl_4_(PPh_3_)_2_, Au_24_Ag_20_(C≡CR)_24_Cl_2_, and Au_43_(C≡CR)_20_/Au_42_Ag_1_(C≡CR)_20_] with similar sizes and core structures but different ligand packing densities to investigate how the number of accessible metal sites impacts CO_2_RR activity and selectivity. We develop a simple method to determine the number of CO_2_-accessible sites for a given NC then use this to probe relationships between surface accessibility and CO_2_RR performance for atomically precise NC catalysts. Specifically, the NCs with the highest number of accessible metal sites [Au_43_(C≡CR)_20_ and Au_42_Ag_1_(C≡CR)_20_] feature a FE_CO_ of >90% at –0.57 V *vs.* the reversible hydrogen electrode (RHE), while NCs with lower numbers of accessible metal sites have a reduced FE_CO_. In addition, CO_2_RR studies performed on other Au–alkynyl NCs that span a wider range of sizes further support the relationship between FE_CO_ and the number of accessible metal sites, regardless of NC size. This work establishes a generalizable approach to evaluating the potential of atomically precise NCs for electrocatalysis.

## Abstract 2

Atomically precise nanoclusters (NCs) provide useful model systems for studying fundamental aspects of the metal-catalysed CO_2_ reduction reaction (CO_2_RR).

## Graphical abstract

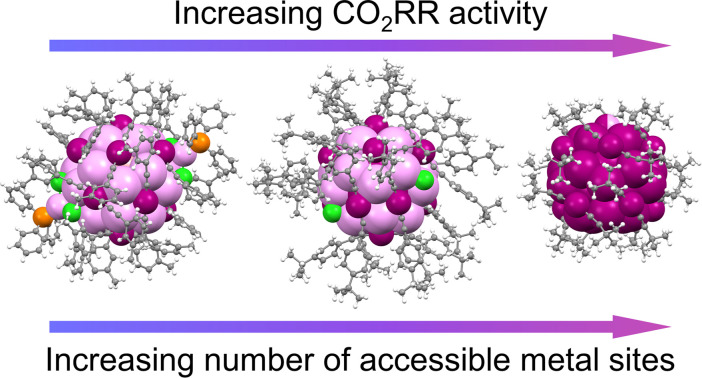

## Introduction

Heterogeneous catalysts composed of metal nanoparticles (NPs) dispersed on high-surface-area supports have been studied for more than a century,^1–3^ and these catalysts are of increasing interest for the electrochemical reduction of CO_2_ into chemical fuels and feedstocks.^4–6^ Gold- and silver-based NPs are particularly effective for the selective reduction of CO_2_ to CO.^7^ Though the effects of nanoparticle size,^8–10^ shape,^11,12^ and surface ligands^13,14^ on the CO_2_ reduction reaction (CO_2_RR) have been widely studied, the nonuniformity of metal NP catalysts is a long-standing challenge in the investigation of fundamental catalytic mechanisms.^1,15^ In particular, it is often difficult to identify the specific active sites that drive catalysis because of the wide distribution of local microenvironments in ligand-protected NPs that adopt varying sizes, shapes, and surface structures.^16,17^ For example, although functionalization with larger organic ligands has been shown to enhance the CO_2_RR activity of Au NPs,^18,19^ uncertainty over the exact arrangement of surface ligands makes it difficult to determine how bulky ligands impact selectivity and catalytic activity. Such molecular-level insight is, however, possible when atomically precise nanoclusters (NCs) are used as catalysts, since their uniformity allows the entire particle structure—including the ligand shell—to be resolved crystallographically.^20–23^

Soon after the canonical Au_25_(SR)_18_ (SR = aryl or alkylthiolate) NC was first reported, it was shown to be effective for CO_2_RR, featuring a high faradaic efficiency for CO (FE_CO_) at –1 V *vs.* the reversible hydrogen electrode (RHE).^24^ Though the CO_2_RR has since been studied for many other atomically precise Au NCs,^25–28^ there still remains much to be understood about how NC size, structure, and surface ligand identity influence catalytic activity and selectivity. For instance, relationships between NC size and CO_2_RR activity are challenging to identify because the ligand-to-metal ratio typically increases for smaller NCs,^29–31^ resulting in higher surface coverage. Changes to the arrangement of surface ligands and metal atoms—as well as the electronic structure of the NC—may also affect the outcome of catalytic reactions, further convoluting structure–property relationships.^32^ Indeed, differing trends have been reported for how NC size affects CO_2_RR activity. For example, in the series Au_25_(SR)_18_, Au_38_(SR)_24_, and Au_144_(SR)_60_ (SR = SC_2_H_4_Ph), CO_2_RR activity increases with increasing NC size,^33^ while other studies have found that the FE_CO_ of Au-SR NCs is not directly affected by NC size.^34^ Given the different size-dependent trends that have been observed for atomically precise NCs, the number of active sites is often a better predictor of catalytic behavior but is difficult to manipulate in a predictable fashion.

In an effort to decouple the role of metal active sites from NC size, structure, and ligand type, we designed a series of alkynyl-protected atomically precise Au/Ag NCs with similar sizes and core structures but different degrees of surface ligand coverage. We investigated the CO_2_RR performance of these NCs and developed a convenient computational method to quantitatively evaluate the accessibility of potential catalytically active sites. Critically, the use of acetylene-based ligands—bearing one rotatable bond—simplifies the conformational landscape at the metal–ligand interface, thereby clarifying the effect of ligand modification on surface coverage and the number of accessible metal sites. In particular, we found that the number and accessibility of surface metal sites is directly correlated to experimental CO_2_RR activity.

## Results and discussion

### Synthesis and characterization of alkynyl-protected Au/Ag NCs

**Fig. 1:**
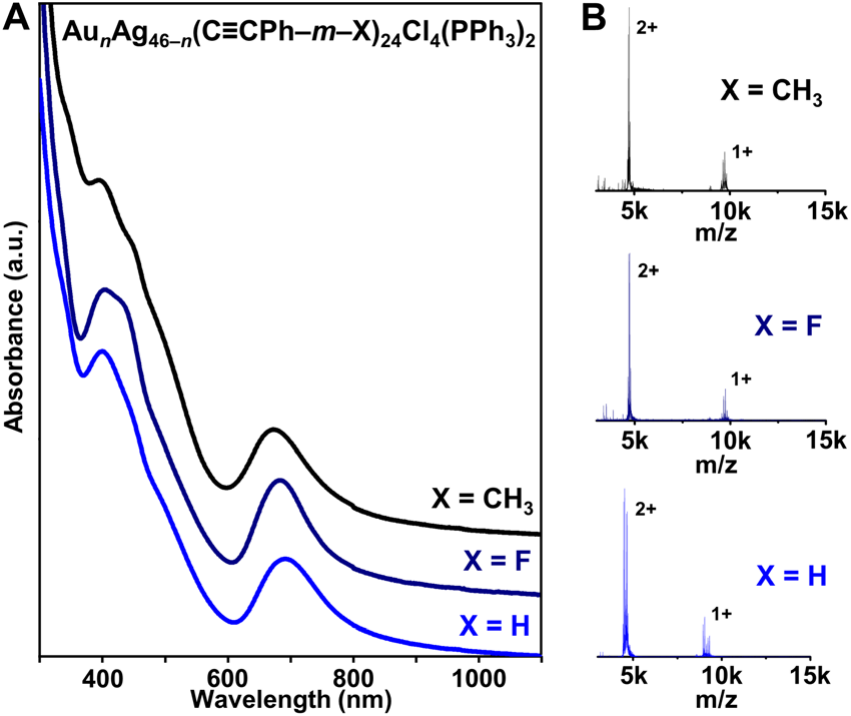
(A) UV-vis-NIR absorption spectra and (B) ESI-MS of Au_*n*_Ag_46−*n*_(C≡CPh–*m*–X)_24_Cl_4_(PPh_3_)_2_ (*n* = 16–19, X = H, F, or CH_3_).

**Fig. 2:**
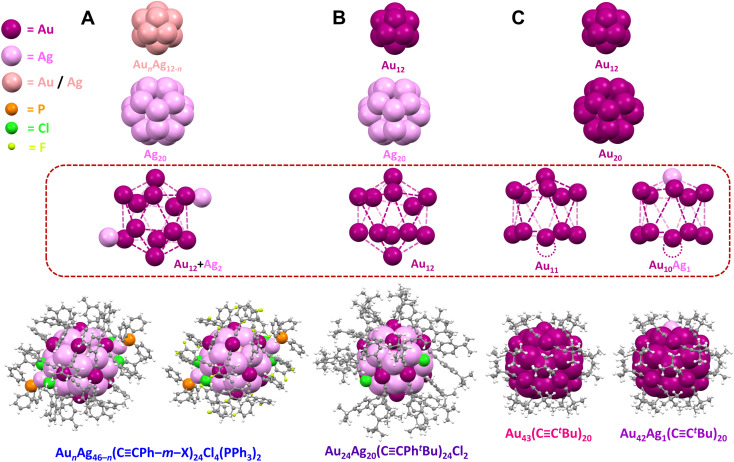
Structures of (A) Au_*n*_Ag_46−*n*_(C≡CPh–*m*–X)_24_Cl_4_(PPh_3_)_2_ (*n* = 16–19, X = F or CH_3_), (B) Au_24_Ag_20_(C≡CPh^*t*^Bu)_24_Cl_2_, and (C) Au_43_(C≡C^*t*^Bu)_20_ and Au_42_Ag_1_(C≡C^*t*^Bu)_20_, Color code: magenta = Au; violet = Ag; light pink = Au/Ag; orange = P; green = Cl; light green = F; grey = C; white = H. The vacant surface sites in Au_43_(C≡C^*t*^Bu)_20_ and Au_42_Ag_1_(C≡C^*t*^Bu)_20_ are indicated with a dashed circle.

We recently reported the isostructural alkynyl-protected NCs Au_43_(C≡C^*t*^Bu)_20_ (Au_43_) and Au_42_Ag_1_(C≡C^*t*^Bu)_20_ (Au_42_Ag_1_), which are synthesized by reducing an oligomeric Au^I^–C≡C^*t*^Bu or Au^I^/Ag^I^–C≡C^*t*^Bu precursor with borane *tert*-butylamine then purifying *via* thin layer chromatography (Fig. S1A†).^35^ With a nearly identical NC core but an increased density of alkynyl surface ligands, we also selected the previously reported Au_24_Ag_20_(C≡CPh^*t*^Bu)_24_Cl_2_ NC (Au_24_Ag_20_) for comparison (Fig. S1B†).^36,37^ To complete a series of NCs with similar sizes and varying surface ligand densities, we also targeted an Au/Ag–alkynyl NC with an even denser organic shell. This was achieved by introducing a bulky triphenylphosphine (PPh_3_) ligand through the “hydride-mediated conversion” method.^38,39^ Specifically, [Au_9_(PPh_3_)_8_]^3+^ (ref. 40) was reduced with NaBH_4_ to furnish a hydride-doped [HAu_9_(PPh_3_)_8_]^2+^ cluster, which was then reacted with CH_3_COOAg, *meta*-substituted phenylacetylene ligands, and triethylamine to yield NCs with a composition of Au_*n*_Ag_46−*n*_(C≡CPh–*m*–X)_24_Cl_4_(PPh_3_)_2_ (Au*_n_*Ag_46−_*_n_*, *n* = 16–19, X = H, F, CH_3_) (Fig. 1, see ESI† for experimental details). Though attempts to grow single crystals of Au_*n*_Ag_46−*n*_(C≡CPh)_24_Cl_4_(PPh_3_)_2_ suitable for structure determination were unsuccessful, crystal structures were successfully determined for Au*_n_*Ag_46−_*_n_* with X = F and CH_3_ (see Fig. 2). The successful crystallization of Au*_n_*Ag_46−_*_n_* with *meta*-substituted phenylacetylene ligands can be attributed to the additional interparticle C–H⋯π, or C–F⋯π interactions between the *meta* functional groups on one NC and the phenyl rings of ligands on another NC (Fig. S2 and S3†).^41,42^

The solution-phase UV-vis absorption spectra of all three Au*_n_*Ag_46−_*_n_* NCs with different protecting ligands exhibit sharp absorption peaks centered near 400 and 690 nm (Fig. 1A). Moreover, electrospray ionization mass spectrometry (ESI-MS) analysis shows 2+ and 1+ ion peaks for the NCs (Fig. 1B). Combined with the X-ray crystallography data, the 2+ and 1+ charges are attributed to ionization in ESI-MS—not the native charge states of the NCs—and the peaks correspond to NCs that have lost one or two PPh_3_ ligands, which has been commonly observed for similar atomically precise NCs (Fig. S4–S6†).^43,44^ The ESI-MS spectrum of Au_*n*_Ag_46−*n*_(C≡CPh–*m*–F)_24_Cl_4_(PPh_3_)_2_ contains peaks corresponding to *n* = 16, 17, and 18 (Fig. S5†), consistent with the crystallographically refined composition of Au_17.67_Ag_28.33_(C≡CPh–*m*–F)_24_Cl_4_(PPh_3_)_2_ (Tables S1 and S2†). When HC≡CPh–*m*–CH_3_ was used instead, three peaks corresponding to *n* = 17, 18, and 19 were found, which is consistent with the higher refined Au : Ag ratio in Au_19_Ag_27_(C≡CPh–*m*–CH_3_)_24_Cl_4_(PPh_3_)_2_ (Tables S3 and S4†). The small inconsistency between MS and X-ray crystallography data has also been observed for other heterometallic NCs, which are known to be dynamic—and prone to rearrangement—in solution.^45,46^ Regardless, the similar UV-vis and ESI-MS spectra for Au_*n*_Ag_46−*n*_(C≡CPh)_24_Cl_4_(PPh_3_)_2_ and Au_*n*_Ag_46−*n*_(C≡CPh–*m*–X)_24_Cl_4_(PPh_3_)_2_ (X = F, CH_3_) NCs—along with the fact that the same synthesis conditions were used—suggest that the three NCs are isostructural. Note that small differences in absorption features near 400 nm and shifts in MS can be attributed to the different alkynyl ligands used.

The series of five atomically precise Au/Ag NCs exhibit similar core structures. Specifically, Au*_n_*Ag_46−_*_n_* has an icosahedral Au_*n*_Ag_12−*n*_ kernel (*n* = 4–7 with Au and Ag randomly distributed), a dodecahedral Ag_20_ inner shell, and an icosahedral Au_12_ outer shell with two additional Ag atoms on the surface (Fig. 2A). Each of the additional Ag atoms on the surface of Au*_n_*Ag_46−_*_n_* is bonded to two chloride ligands, one Au atom in the outer shell, and one PPh_3_ ligand. Four chloride ligands are necessary for Au*_n_*Ag_46−_*_n_* to adopt a closed-shell superatomic electronic configuration (46 − 24 − 4 = 18 e^–^) similar to Au_24_Ag_20_ with two chloride ligands (24 + 20 − 24 − 2 = 18 e^–^). The structure of Au_24_Ag_20_ also consists of an icosahedral Au_12_ kernel, a dodecahedral Ag_20_ inner shell, and an icosahedral Au_12_ outer shell (Fig. 2B), while Au_43_/Au_42_Ag_1_ has an icosahedral Au_12_ kernel, a dodecahedral Au_20_ inner shell, and an incomplete icosahedral Au_11_ or Au_10_Ag_1_ outer shell with a single vacant surface site (Fig. 2C).

Adding or removing a single metal atom to or from the surface of NCs has provided insight into the optical and electronic properties of thiolate-capped metal NCs.^47,48^ The successful synthesis of our series of NCs with the same core (M_46_, M_44_, and M_43_; M = Au and/or Ag) represents—to the best of our knowledge— the first demonstration of atom-by-atom evolution for alkynyl-stabilized metal NCs structure (Fig. 2). Moreover, the linear directionality of alkynyl ligands in alkynyl-stabilized NCs offer advantages for catalytic studies since the NC/electrolyte interface is dominated by Au–C≡C bonds with similar local arrangements. Note that for Au*_n_*Ag_46−_*_n_* and Au_24_Ag_20_, Ag atoms are located in the inner dodecahedral Ag_12_ shell and/or the icosahedral Au_*n*_Ag_12−*n*_ kernel which are not accessible to substrates interacting with the surface of the NC (Fig. 2, highlighted by the dashed red box). Importantly, the surface ligand density varies systematically across the series: 24 alkynyl, 4 chloride, and 2 phosphine ligands for Au*_n_*Ag_46−_*_n_*, 24 alkynyl and 2 chloride ligands for Au_24_Ag_20_, and only 20 alkynyl ligands for Au_43_ and Au_42_Ag_1_. Thus, this series of atomically precise NCs provides a powerful platform to study relationships between metal site accessibility and CO_2_RR activity.

### Evaluation of CO_2_RR activity and selectivity

**Fig. 3:**
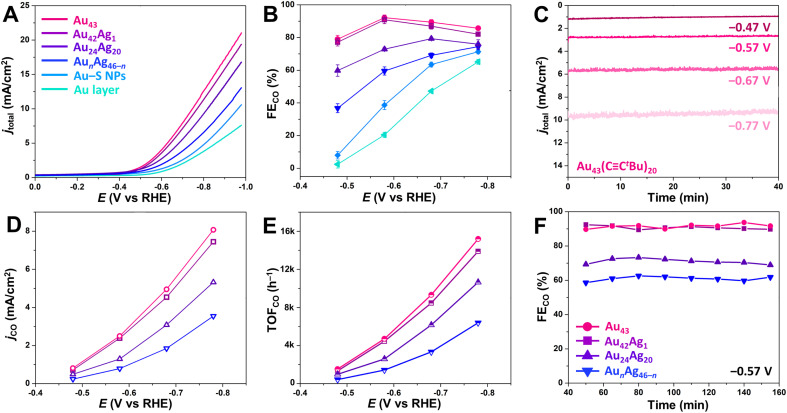
(A) LSV and (B) FE_CO_ for CO_2_RR for the series of NC-based catalysts. (C) Chronoamperometry data for the Au_43_-based catalyst at different applied potentials. (D) *j*_CO_ and (E) TOF_CO_ for the NC-based catalysts at different applied potentials during CO_2_RR. (F) FE_CO_ for the NC-based catalysts at –0.57 V *vs.* RHE during CO_2_RR over an extended time period. All experiments were conducted in a 0.5 M KHCO_3_ solution saturated with CO_2_. In panels A, B, D, E, and F, magenta represents Au_43_(C≡C^*t*^Bu)_20_, purple represents Au_42_Ag_1_(C≡C^*t*^Bu)_20_, indigo represents Au_24_Ag_20_(C≡CPh^*t*^Bu)_24_Cl_2_, blue represents Au_*n*_Ag_46−*n*_(C≡CPh)_24_Cl_4_(PPh_3_)_2_, sky blue represents Au-SC_2_H_4_Ph NPs, and cyan represents the Au layer.

To investigate their efficacy for CO_2_RR catalysis, synthesized atomically precise NCs were mixed with carbon black (20 wt% NC loading) and deposited on a carbon paper electrode. For comparison, a carbon electrode was also prepared with spherical Au–SC_2_H_4_Ph NPs (Au–S NPs) with an average diameter of 3.1 ± 0.4 nm (Fig. S1C†) that were mixed with carbon black at the same mass loading. To provide an additional comparison, an 85 nm thick bulk gold layer was also deposited on one carbon paper electrode (referenced as Au layer) by electron beam deposition. Linear sweep voltammetry (LSV) was then performed for each electrode in CO_2_-saturated 0.5 M KHCO_3_ solution. For the atomically precise NCs, the current density (*j*_total_) was found to increase in the order of Au*_n_*Ag_46−_*_n_* < Au_24_Ag_20_ < Au_42_Ag_1_ < Au_43_ (Fig. 3A). Larger sized Au–S NPs exhibited a lower *j*_total_ than all atomically precise NCs, and *j*_total_ for the Au layer was the lowest of all catalysts investigated here (Fig. 3A). We note that the NC catalysts were not activated before electrocatalysis to minimize possible ligand stripping, and the LSV curves taken before and after chronoamperometric CO_2_RR catalysis (potential range of –0.47 V to –0.77 V *vs.* RHE) were in close agreement (Fig. S7†). Moreover, the NC-based catalysts display a steady current density over at least 40 min at each applied voltage (–0.47 V, –0.57 V, –0.67 V and –0.77 V *vs.* RHE; Fig. 3C), and the absorption spectra of NCs recovered from the electrode after catalysis matched those of as-synthesized NCs (Fig. S8†), confirming the stability of the NCs during CO_2_RR catalysis.

For all atomically precise NC catalysts evaluated here, CO was the major CO_2_RR product, and H_2_ was the sole byproduct with no liquid products detected by ^1^H NMR spectroscopy. The faradaic efficiency for CO production (FE_CO_) was assessed for each catalyst by calculating the percentage of transferred charge that was directed toward CO production (see ESI† for details), and the highest FE_CO_ were 92.1 ± 1.7% and 90.9 ± 1.4% for Au_43_ and Au_42_Ag_1_, respectively, at a potential of −0.57 *vs.* RHE (Fig. 3B). At the same potential, FE_CO_ for Au_24_Ag_20_ and Au*_n_*Ag_46−_*_n_* were just 72.9 ± 1.0% and 59.5 ± 2.5%, respectively (Fig. 3B). These lower efficiencies can be attributed to more densely packed surface ligands, which likely favors the hydrogen evolution reaction (HER) over CO_2_RR.^25,26,49^ The Au–S NPs have an even lower FE_CO_ (38.7 ± 0.8%) at the same potential, which is consistent with previously reported studies.^27,33^ If FE_CO_ is related to the density of surface ligands, one might assume that a ligand-free Au layer would have the highest efficiency. However, the FE_CO_ for the Au layers is only 20.4 ± 1.6% at –0.57 V *vs.* RHE, highlighting the important role of the microenvironments created by nanostructured catalysts in driving CO_2_RR.^3,50,51^

To further evaluate catalytic performance, CO partial current densities (*j*_CO_) were determined and compared across the NC series. Generally, *j*_CO_ decreases with increasing ligand density on the surface (Fig. 3D): Au_43_/Ag_42_Ag_1_ (20 surface ligands) > Au_24_Ag_20_ (26 surface ligands) > Au*_n_*Ag_46−_*_n_* (30 surface ligands). For Au_43_, *j*_CO_ is slightly higher than for Au_42_Ag_1_, suggesting that substitution of a single surface Au atom for Ag leads to a small decrease in CO_2_RR performance, particularly at more negative potentials. Furthermore, Au_43_ shows a high CO turnover frequency (TOF_CO_) of 4718 h^−1^ at −0.57 V and 15 193 h^−1^ at −0.77 V *vs.* RHE (Fig. 3E), which exceeds the values for Au_24_Ag_20_ (2597 h^−1^ at −0.57 V and 10 658 h^−1^ at −0.77 V) and Au*_n_*Ag_46−_*_n_* (1427 h^−1^ at −0.57 V and 6400 h^−1^ at −0.77 V). Note that the same mass loading of NCs was used for all experiments. This makes it reasonable to directly compare TOF values since the NCs in this series have similar molecular weights (Table S5†). In addition, differences in catalytic activity cannot be attributed to differences in NC stability as the FE_CO_ of all catalysts remained constant for at least 2.5 hours at −0.57 V (Fig. 3F).

While the nature of active sites is regarded as one of the best predictors of catalytic activity,^52^ it is often challenging to experimentally determine the number of catalytically active sites in a nanostructured material, and theoretical models are required.^53,54^ Though double-layer capacitance measurements can be used to determine the electrochemically active surface area (ECSA) of catalysts, the ECSA might not reflect the surface area active specifically for CO_2_RR since CO_2_RR and HER frequently occur simultaneously. For example, previous studies have shown that Au_25_(SR)_18_ and Au_38_(SR)_24_ exhibit different CO_2_RR behavior even though the NCs have almost the same ECSA.^33,49^ The ligand-to-metal ratio can serve as a proxy for active site density when the sizes of NCs are similar,^55^ but this ratio is not directly related to the number of active sites owing to the different shapes and surface structures that similarly sized NCs can adopt. Indeed, two isomeric Au_38_(SR)_24_ NCs with the same ligand-to-metal ratio have shown significant differences in CO_2_RR catalysis.^34^

**Fig. 4:**
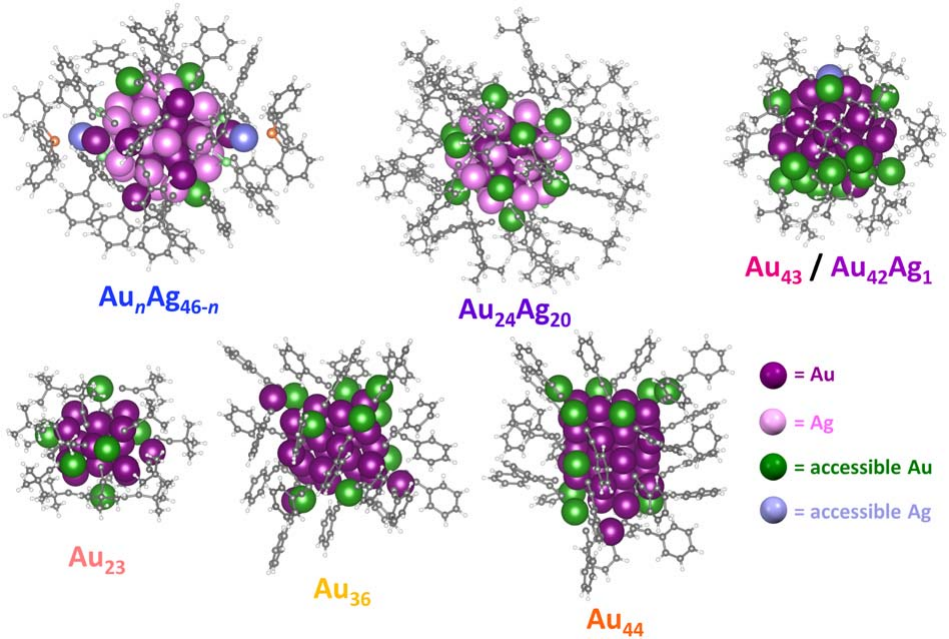
The metal sites accessible to CO_2_ on the surface of different Au/Ag-alkynyl NCs.

With these challenges in mind, we sought to establish a simple method for determining the number of metal sites accessible to CO_2_ in atomically precise NCs that does not rely on computationally intensive density functional theory (DFT) calculations. Briefly, the NC structure determined by crystallography is used to generate a series of several thousand conformers accounting for the different ligand conformations that may arise due to ligand rotation in the absence of crystal packing effects (see ESI† for details). The accessible surface area of each conformation within the conformer series was then calculated for every surface atom using a 1.65 Å spherical probe (the kinetic radius of CO_2_, Fig. S9†).^56^ Metal atoms with a positive contact area with CO_2_ (Fig. S10†) were counted as accessible since these atoms have sufficient space to accommodate a covalent bond with CO_2_. Accessible metal atoms for the different alkynyl-protected NCs under investigation are highlighted in Fig. 4. Note that the NC conformation with the greatest number of accessible metal atoms (*N*) was used to represent the surface accessibility of each NC. Using this approach, the number of accessible metal atoms for each NC can be calculated in ∼1 hour. Notably, the number of accessible metal atoms, *N*, for Au_43_/Au_42_Ag_1_, Au_24_Ag_20_, and Au*_n_*Ag_46−_*_n_* are 16, 12, and 5, respectively, which is consistent with a greater density of CO_2_-accessible surface metal sites driving increased CO_2_RR activity. The role of CO_2_-accessible metal sites is further supported by the fact that we observe little variation in TOF_CO_ when it is normalized to the number of accessible metal sites on each NC (TOF_CO_/*N*). Specifically, TOF_CO_/*N* for Au_43_, Au_42_Ag_1_, Au_24_Ag_20_, and Au*_n_*Ag_46−_*_n_* is 294 h^−1^, 279 h^−1^, 216 h^−1^ and 285 h^−1^, respectively, at –0.57 V *vs.* RHE (Table 1 and Fig. S11†). This suggests that the number of CO_2_-accessible metal sites—rather than the degree of Ag doping,^57^ surface ligand functional groups,^58^ or the electronic structure^59^ of the cluster—is the primary driver of catalytic activity, at least for NCs with relatively similar structures and compositions.

**Table 1:** The calculated number of accessible metal sites (*N*) for a series of alkynyl-protected NCs, along with their experimentally determined FE_CO_, *j*_CO_, TOF_CO_, and TOF_CO_/*N* values for CO_2_RR electrocatalysis at −0.57 V *vs.* RHE. All experiments were conducted in a 0.5 M KHCO_3_ solution saturated with CO_2_

Catalyst	Number of accessible metals (*N*)	FE_CO_ (%)	*j*_CO_ (mA cm^−2^)	TOF_CO_ (h^−1^)	TOF_CO_/*N* (h^−1^)
Au_43_	16	92.1 ± 1.7	2.5	4718	295
Au_42_Ag_1_	16	90.9 ± 1.4	2.4	4458	279
Au_24_Ag_20_	12	72.9 ± 1.0	1.3	2597	216
Au_*n*_Ag_46−*n*_	5	59.5 ± 2.5	0.8	1427	285
Au_44_	12	74.5 ± 1.3	1.0	2045	170
Au_36_	9	69.7 ± 1.6	0.7	1325	147
Au_23_	6	66.7 ± 2.5	0.7	794	132

**Fig. 5:**
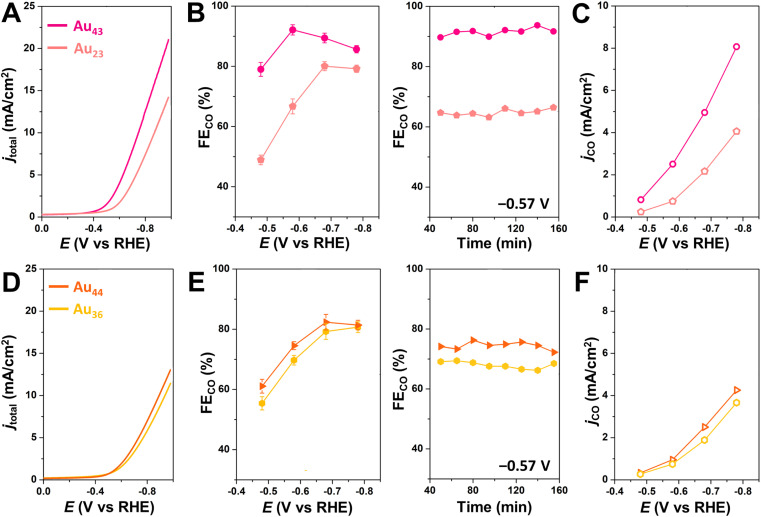
(A/D) LSV, (B/E) FE_CO_ at different applied potentials (left), or at –0.57 V *vs.* RHE for prolonged time (right), and (C/F) *j*_CO_ at different applied potentials during CO_2_RR for NC-based catalysts conducted in CO_2_-saturated 0.5 M KHCO_3_ solution. Magenta represents Au_43_(C≡C^*t*^Bu)_20_, pink represents Au_23_(C≡C^*t*^Bu)_20_, orange represents Au_44_(C≡CPh)_28_, and yellow represents Au_36_(C≡CPh)_24_.

To further investigate the generalizability of accessible metal site number as a predictor of CO_2_RR activity, we also evaluated a wider range of previously reported alkynyl-protected Au NCs: Au_23_(C≡C^*t*^Bu)_15_ (Au_23_),^60^ Au_36_(C≡CPh)_24_ (Au_36_), and Au_44_(C≡CPh)_28_ (Au_44_)^61^ (Fig. S12†). Within this series, Au_44_ has the highest number of CO_2_-accessible metal sites (*N* = 12) and the highest CO_2_RR activity, while Au_23_ has the lowest number of CO_2_-accessible sites and the lowest CO_2_RR activity (Fig. 5). The activity of Au_44_, however, is much lower than that of Au_43_, which is consistent with the greater number of CO_2_-accessible metal sites (*N* = 16) for the latter NC (Table 1 and Fig. S13†). The relationship between the number of accessible metal sites and FE_CO_, *j*_CO_ is plotted in Fig. S14.† This highlights that even though larger sized Au–alkynyl NCs often have increased CO_2_RR activities, just like their Au–thiolate counterparts,^33^ the number of accessible surface metals tends to be more closely related to catalyst performance. Therefore, we conclude that the number of accessible metal sites provides a useful metric for evaluating the likelihood of CO_2_ binding to a particular atomically precise NC and for predicting trends in catalytic activity. Since Au_43_ and Au_42_Ag_1_ NCs are isostructural, their slight difference in CO_2_RR activity could be due to replacing a surface Au atom with a more electropositive Ag atom.^35^ Though Au_24_Ag_20_ and Au_44_ NCs have the same number of metal atoms (44) as well as the number of accessible metals (12), Au_44_ exhibits a slightly higher FE_CO_ and lower *j*_CO_ than Au_24_Ag_20_. Differences in the geometric and electronic structures of the NCs may influence their CO_2_RR performance, but this is likely a less significant effect than the number of accessible metals. For NCs of the same core structure, more valence electrons in the frontier molecular orbitals elevate the energy of the highest occupied molecular orbital (HOMO), thereby favoring electron transfer from the NC catalyst to the substrate and thus improving the CO_2_RR activity.^59^ The performance of Au_43_ and Au_42_Ag_1_ NCs could also be partially attributed to more facile electron transfer during electrocatalysis. However, since the electronic structure of the NCs is determined by the number of metals and ligands, the surface coverage is still of importance. Moreover, since calculating the number of accessible metal sites is straightforward, it can serve as a quick screening tool for identifying the most promising NCs for electrochemical catalysis that is complementary to advanced DFT calculations.

## Conclusions

A series of alkynyl-stabilized NCs with similar sizes and core structures but different degrees of surface ligand coverage was used to provide insight into the effect of the number of accessible metal sites on electrochemical CO_2_RR activity. A simple computational method was developed to calculate the number of metal sites on the NCs that are accessible to CO_2_. The highest faradaic efficiencies for CO_2_RR were observed for Au_43_ and Au_42_Ag_1_, which feature the largest number of accessible metal sites. When the TOF_CO_ of the NC-based catalysts was normalized by the number of accessible sites, the differences between NCs were reduced. Collectively, these trends suggest that the number of substrate-accessible metal sites serves as a useful and generalizable predictor for evaluating the potential of atomically precise NCs for CO_2_RR.

## Data availability

All the data are shown in the manuscript or the associated ESI.†

## Author contributions

J. A. M and Y. L. conceived the project. Y. L. synthesized the NCs and grew the single crystals. Y. L. and A. E. T. performed the electrochemical studies and analysed the data. G. J. S performed the computational studies. R. D. M. and S.-L. Z. solved the crystal structures. Y. L and G. J. S wrote the manuscript with contribution from other authors.

## Conflicts of interest

There are no conflicts to declare.

## Supplementary Material

SC-014-D3SC04085B-s001

SC-014-D3SC04085B-s002
