# Supplementary material for: The role of metal accessibility on carbon dioxide electroreduction in atomically precise nanoclusters
Source: Chem Sci. 2023 Oct 24;14(43):12283–91. doi: 10.1039/d3sc04085b (PMC10631301; doi:10.1039/d3sc04085b)
Supplement: SC-014-D3SC04085B-s001 [file SC-014-D3SC04085B-s001.pdf]

Electronic Supplementary Material (ESI) for Chemical Science.
This journal is © The Royal Society of Chemistry 2023

Electronic Supplementary Information

# The Role of Metal Accessibility on Carbon Dioxide Electroreduction in Atomically Precise Nanoclusters

*Yingwei Li,*[‡] *Grant J. Stec,*[‡] *Agnes E. Thorarinsdottir, Ryan D. McGillicuddy, Shao-Liang Zheng, and Jarad A. Mason**

*Department of Chemistry & Chemical Biology, Harvard University, 12 Oxford Street, Cambridge, Massachusetts, 02138, United States.*
*E-mail: mason@chemistry.harvard.edu*

*Chem. Sci.*

## Table of Contents

**1. Chemicals**
**2. Synthesis**
2.1 Synthesis of $Au_nAg_{46-n}$(C≡CPh–*m*–X)$_{24}Cl_4(PPh_3)_2$ (*n* = 16–19, X = H, F, $CH_3$) nanoclusters
2.2 Synthesis of $Au_{24}Ag_{20}$(C≡CPh$^t$Bu)$_{24}Cl_2$ nanoclusters
2.3 Synthesis of $Au_{43}$(C≡C$^t$Bu)$_{20}$ nanoclusters
2.4 Synthesis of $Au_{42}Ag_1$(C≡C$^t$Bu)$_{20}$ nanoclusters
2.5 Synthesis of Au–($SC_2H_4Ph$) nanoparticles
2.6 Preparation of gold-coated carbon paper
2.7 Synthesis of $Au_{44}$(C≡CPh)$_{28}$ and $Au_{36}$(C≡CPh)$_{24}$ nanoclusters
**3. Solution-phase characterization**
**4. X-ray crystallography**
**5. Electrochemical measurements**
**6. Computational methods**
**7. Supplementary Tables S1-S5**
**Table S1.** Crystal data for $Au_{17.67}Ag_{28.33}$(C≡CPhF)$_{24}Cl_4(PPh_3)_2$
**Table S2.** Atomic percentages of Au and Ag in the icosahedral $M_{12}$ kernel of $Au_{17.67}Ag_{28.33}$(C≡CPhF)$_{24}Cl_4(PPh_3)_2$ determined by X-ray crystallography
**Table S3.** Crystal data for $Au_{19}Ag_{27}$(C≡CPhCH$_3$)$_{24}Cl_4(PPh_3)_2$
**Table S4.** Atomic percentages of Au and Ag in the icosahedral $M_{12}$ kernel of $Au_{19}Ag_{27}$(C≡CPhCH$_3$)$_{24}Cl_4(PPh_3)_2$ determined by X-ray crystallography
**Table S5.** Molecular weights of the alkynyl-protected NCs.
**8. Supplementary Figures S1-S12**
**Figure S1.** UV-vis-NIR absorption spectra of $Au_{43}$(C≡C$^t$Bu)$_{20}$ and $Au_{42}Ag_1$(C≡C$^t$Bu)$_{20}$ NCs, $Au_{24}Ag_{20}$(C≡CPh$^t$Bu)$_{24}Cl_2$ NCs, and Au–thiolate NPs
**Figure S2.** Interparticle ligand interactions between two $Au_nAg_{46-n}$(C≡CPh–*m*–F)$_{24}Cl_4(PPh_3)_2$ NCs
**Figure S3.** Interparticle ligand interactions between two $Au_nAg_{46-n}$(C≡CPh–*m*–CH$_3$)$_{24}Cl_4(PPh_3)_2$ NCs
**Figure S4.** ESI-MS spectra of $Au_nAg_{46-n}$(C≡CPh)$_{24}Cl_4(PPh_3)_2$
**Figure S5.** ESI-MS spectra of $Au_nAg_{46-n}$(C≡CPh–*m*–F)$_{24}Cl_4(PPh_3)_2$
**Figure S6.** ESI-MS spectra of $Au_nAg_{46-n}$(C≡CPh–*m*–CH$_3$)$_{24}Cl_4(PPh_3)_2$
**Figure S7.** LSV before and after chronoamperometric $CO_2$RR catalysis for $Au_{43}$(C≡C$^t$Bu)$_{20}$, $Au_{42}Ag_1$(C≡C$^t$Bu)$_{20}$, $Au_{24}Ag_{20}$(C≡CPh$^t$Bu)$_{24}Cl_2$, and $Au_nAg_{46-n}$(C≡CR)$_{24}Cl_4(PPh_3)_2$
**Figure S8.** UV-vis-NIR absorption spectra of $Au_{43}$(C≡C$^t$Bu)$_{20}$, $Au_{42}Ag_1$(C≡C$^t$Bu)$_{20}$, $Au_{24}Ag_{20}$(C≡CPh$^t$Bu)$_{24}Cl_2$, and $Au_nAg_{46-n}$(C≡CPh)$_{24}Cl_4(PPh_3)_2$ NCs before (colored lines) and after (black lines) electrolysis
**Figure S9**. Illustration of accessible surface area calculation by the Shrake-Rupley method using the kinetic radius of $CO_2$ (1.65 Å) as a probe.
**Figure S10.** The plot of the number of accessible metal sites of different NCs to the different threshold area
**Figure S11.** Potential-dependent $TOF_{CO}$ and $TOF_{CO}/N$ of NC-based catalysts. NCs include $Au_{43}$(C≡C$^t$Bu)$_{20}$, $Au_{42}Ag_1$(C≡C$^t$Bu)$_{20}$, $Au_{24}Ag_{20}$(C≡CPh$^t$Bu)$_{24}Cl_2$, and $Au_nAg_{46-n}$(C≡CPh)$_{24}Cl_4(PPh_3)_2$. *N* is the number of accessible metal sites of each NC

**Figure S12.** UV-vis-NIR absorption spectra for $Au_{23}(C{\equiv}C^tBu)_{15}$, $Au_{44}(C{\equiv}CPh)_{28}$, and $Au_{36}(C{\equiv}CPh)_{24}$ NCs

**Figure S13.** Potential-dependent $TOF_{CO}$ and $TOF_{CO}/N$ of NC-based catalysts. NCs include $Au_{43}(C{\equiv}C^tBu)_{20}$, $Au_{23}(C{\equiv}C^tBu)_{15}$, $Au_{44}(C{\equiv}CPh)_{28}$, and $Au_{36}(C{\equiv}CPh)_{24}$. $N$ is the number of accessible metal sites of each NC

**Figure S14.** The relationship between the number of accessible metal sites and $FE_{CO}$ and $j_{CO}$

**9. Supporting references**

## 1. Chemicals

(Dimethylsulfide)gold(I) chloride ($(CH_3)_2SAuCl$, 97%, TCI), chloro(triphenylphosphine)gold(I) ($(Ph_3P)AuCl$, ≥99.9% trace metals basis, Aldrich), gold(III) chloride trihydrate ($HAuCl_4·3H_2O$, ≥99.9% trace metals basis, Aldrich), silver acetate ($CH_3COOAg$, 99.99% trace metals basis, Aldrich), silver nitrate ($AgNO_3$, >99%, Aldrich), triethylamine ($Et_3N$, 99.5%, Aldrich), 3,3-dimethyl-1-butyne ($HC≡C^tBu$, 98%, Alfa Aesar), 4-*tert*-butylphenylacetylene ($HC≡CPh^tBu$, 96%, Aldrich), phenylacetylene (HC≡CPh, 98%, Aldrich), 1-ethynyl-3-fluorobenzene (HC≡CPh-*m*-F, 98%, Aldrich), *m*-tolylacetylene ($HC≡CPh$-*m*-$CH_3$, 97%, Thermo Scientific), 2-phenylethanethiol ($HSC_2H_4Ph$, 99%, Aldrich), tetraoctylammonium bromide (TOABr, 98%, Aldrich), borane *tert*-butylamine complex ($(CH_3)_3CNH_2·BH_3$, 97%, Aldrich), sodium borohydride ($NaBH_4$, >98%, Aldrich), tetrahydrofuran (HPLC grade), chloroform ($CHCl_3$, HPLC grade), methanol ($CH_3OH$, HPLC grade), ethanol ($CH_3CH_2OH$, HPLC grade), dichloromethane ($CH_2Cl_2$, HPLC grade), *n*-hexane (HPLC grade), acetonitrile ($CH_3CN$, HPLC grade), potassium hydrogen carbonate ($KHCO_3$, Aldrich), thin-layer chromatography (TLC) plates (SiliCycle SiliaPlate™), carbon black (Vulcan XC 72, Fuel Cell Store), and non-woven carbon paper containing a microporous layer treated with polytetrafluoroethylene to 5 wt % (Sigracet 22 BB, Fuel Cell Store) were used as received.

## 2. Synthesis

**2.1 Synthesis of $Au_nAg_{46-n}(C≡CPh–m–X)_{24}Cl_4(PPh_3)_2$ (*n* = 16–19, X = H, F, CH₃) nanoclusters.** $[Au_9(PPh_3)_8](NO_3)_3$ NCs were synthesized in high yield according to literature procedures[s1] with some modification. 494.7 mg of $(Ph_3P)AuCl$ (1 mmol) dissolved in 10 mL of $CH_2Cl_2$ was added dropwise to a 30 mL methanol solution containing 339.7 mg of $AgNO_3$ (2 mmol). After stirring at room temperature for 15 min, the suspension was filtered, and the clear solution was rotary evaporated to yield a white solid. The white solid was extracted with $CH_2Cl_2$ and dried under vacuum overnight to yield pure $(Ph_3P)AuNO_3$. 469.2 mg of $(Ph_3P)AuNO_3$ (0.9 mmol) was dissolved in 20 mL of ethanol, and 8.7 mg of $NaBH_4$ (0.23 mmol) dissolved in 10 mL of ethanol was added dropwise. After reacting for 2 h, the solvent was rotary evaporated, and the resulting orange solid was extracted by 3 mL of $CH_2Cl_2$ and dried again. The residue was washed with tetrahydrofuran and *n*-hexane several times until the supernatant was clear. The resulting dark green solid was determined to be $[Au_9(PPh_3)_8](NO_3)_3$.

20.3 mg of $[Au_9(PPh_3)_8](NO_3)_3$ (5.0 μmol) was dissolved in 5 mL of $CH_2Cl_2$, and the orange solution was cooled in an ice water bath. 50 μL of an ethanolic solution of $NaBH_4$ (0.10 M) was added, and 30 s later, 2 mL acetonitrile solution containing 10.2 mg $AgNO_3$ of (0.06 mmol), 10 μL of HC≡CPh or 10.5 μL of HC≡CPh-*m*-F or 11.6 μL of $HC≡CPh$–*m*–$CH_3$ (0.09 mmol) and 12.5 μL of $Et_3N$ (0.09 mmol) was added dropwise. The reaction was stirred at room temperature for 4–6 h before the solvent was rotary evaporated. The solids were thoroughly washed by methanol, and the **$Au_nAg_{46-n}(C≡CPh–m–X)_{24}Cl_4(PPh_3)_2$** NCs were extracted by $CH_2Cl_2$ for characterization.

Plate-shaped single crystals of $Au_nAg_{46-n}(C≡CPh–m–F)_{24}Cl_4(PPh_3)_2$ and $Au_nAg_{46-n}(C≡CPh–m–CH_3)_{24}Cl_4(PPh_3)_2$ NCs were obtained via diffusion of acetonitrile into a $CH_2Cl_2$ solution of the NCs at 4 °C for one week.

**2.2 Synthesis of $Au_{24}Ag_{20}(C{\equiv}CPh^tBu)_{24}Cl_2$ nanoclusters.** $Au_{24}Ag_{20}(C{\equiv}CPh^tBu)_{24}Cl_2$ NCs were prepared according to previous reports[s2,s3] with some modification. 29.5 mg of $(CH_3)_2SAuCl$ (0.1 mmol) and 25 mg of $CH_3COOAg$ (0.15 mmol) were dissolved in a mixed solvent containing 10 mL of $CH_2Cl_2$ and 5 mL of methanol. The solution was cooled to 0 °C in an ice water bath and kept stirring for 5 min. Then, 100 μL of $Et_3N$ and 50 μL of $HC{\equiv}CPh^tBu$ in 1 mL of methanol was added to the mixture, followed by dropwise addition of 25 mg of $(CH_3)_3CNH_2{\cdot}BH_3$ dissolved in 2 mL of $CH_2Cl_2$. The reaction was stirred at room temperature overnight. After rotary evaporating the solvent, the **$Au_{24}Ag_{20}(C{\equiv}CPh^tBu)_{24}Cl_2$** NCs were washed thoroughly with methanol, and extracted with $CH_2Cl_2$ for characterization (Figure S1A).

**2.3 Synthesis of $Au_{43}(C{\equiv}C^tBu)_{20}$ nanoclusters.** 58.9 mg of $(CH_3)_2SAuCl$ (0.2 mmol) was dissolved in a mixed solvent containing 4 mL of THF and 9 mL of $CHCl_3$ with 45 μL of $HC{\equiv}C^tBu$ and 40 μL of $Et_3N$ for 15 min. Then, 3 mg of $(CH_3)_3CNH_2{\cdot}BH_3$ dissolved in 1 mL methanol was added at once, and the reaction was continued overnight at room temperature. After rotary evaporating the solvent, the resulting mixture of Au nanoclusters was washed thoroughly with methanol, extracted with $CH_2Cl_2$, and concentrated for TLC separation. The mixture of nanoclusters was pipetted onto the TLC plate, and the separation was conducted in the developing chamber (solvent 1:1 (v/v) $CH_2Cl_2$:*n*-hexane) for 30 min. The first and second bands corresponding to **$Au_{43}(C{\equiv}C^tBu)_{20}$** and **$Au_{23}(C{\equiv}C^tBu)_{15}$** NCs, respectively, were collected and dissolved in $CH_2Cl_2$ for characterization (Figure S1B, Figure S11A).

**2.4 Synthesis of $Au_{42}Ag_1(C{\equiv}C^tBu)_{20}$ nanoclusters. $Au_{42}Ag_1(C{\equiv}C^tBu)_{20}$** NCs were obtained by using 56 mg of $(CH_3)_2SAuCl$ and 1.59 mg of $CH_3COOAg$ (molar ratio Au:Ag = 20:1), while other conditions and separation procedures as detailed above for the synthesis of **$Au_{43}(C{\equiv}C^tBu)_{20}$** were kept the same (Figure S1B).

**2.5 Synthesis of Au–($SC_2H_4Ph$) nanoparticles.** A 15 mL aqueous solution of $HAuCl_4{\cdot}3H_2O$ (30 mM) was mixed with a 40 mL toluene solution of TOABr (50 mmol/L). The two-phase mixture was vigorously stirred until all the gold salt was phase-transformed to the organic phase. After removing the aqueous phase, 0.42 mmol of $HSC_2H_4Ph$ was added. 15 min later, 12.5 mL of a freshly prepared ice-cold aqueous solution of $NaBH_4$ (0.4 M) was added with vigorous stirring. The reaction was stirred at room temperature overnight. After reducing the volume of the solvent to 10 mL by rotary evaporation, excess methanol was added and the mixture was centrifuged at 8000 rpm for 5 min to precipitate the nanoparticles. The precipitate was washed with methanol 3 more times, and the nanoparticles were extracted with $CH_2Cl_2$ (Figure S1C) for characterization.

**2.6 Preparation of gold-coated carbon paper.** Gold was deposited onto a non-woven carbon paper containing a microporous layer (treated with polytetrafluoroethylene to 5 wt %) using electron beam deposition tool (Denton EE-4). 85 nm thick layer of gold was deposited at a deposition rate of 2 Å per second.

**2.7 Synthesis of $Au_{44}(C{\equiv}CPh)_{28}$ and $Au_{36}(C{\equiv}CPh)_{24}$ nanoclusters.** $Au_{44}(C{\equiv}CPh)_{28}$ and $Au_{36}(C{\equiv}CPh)_{24}$ NCs were synthesized according to previous reports (Figures S11B and S11C).[s4]

## 3. Solution-phase characterization

UV-vis-NIR spectra were obtained with a Cary 5000 spectrometer. ESI mass spectrometry spectra were recorded using a Waters quadrupole time-of-flight (QTOF) mass spectrometer equipped with a Z-Spray Source. Scanning transmission electron microscope images were obtained on a JEOL JEM2100F STEM.

## 4. X-ray crystallography

Suitable crystals were mounted onto a MiTeGen capillary with fluorolube and measured on a Bruker D8 VENTURE diffractometer equipped with a PHOTON III-C14 detector and $MoK_\alpha$ X-ray source ($\lambda$ = 0.71073 Å). The crystals were kept at 100 K during data collection. Using Olex2,[s5] the structures were solved with the olex2.solve[s6] structure solution program using charge flipping and refined with the ShelXL[s7] refinement package using least squares minimization. All Au, Ag and C atoms were found directly. All non-hydrogen atoms were refined anisotropically. All H atoms were set in geometrically calculated positions and refined isotropically using a riding model. Refinement details can be found in each CIF.

## 5. Electrochemical measurements

The catalyst ink was prepared by dispersing 2 mg of different NCs or NPs, 8 mg of carbon black (20 wt %), and 20 µL of Nafion solution (5 wt % in a mixture of lower aliphatic alcohols and water) in 1 mL mixture of toluene, dichloromethane and ethanol (v : v : v = 2 : 7 : 1) by sonication for 30 min to form a homogeneous ink. 50 µL of the homogeneous ink was loaded onto a carbon paper electrode of 1×1 $cm^2$ geometric surface area. Each electrode was dried in air overnight prior to use.

All electrochemical measurements were carried out in a custom gas-tight H-cell with two compartments separated by a Nafion 117 membrane. A 0.5 M $KHCO_3$ aqueous solution was used as the electrolyte, and each of the H-cell compartments was filled with 100 ml of electrolyte solution, leaving ~10 ml of headspace. A CH Instruments potentiostat (model 760C) was used for the electrochemical $CO_2$ reduction reaction ($CO_2$RR) experiments with the catalyst-loaded carbon paper as a working electrode. An Ag/AgCl electrode (leakless) and a platinum mesh were used as the reference and counter electrodes, respectively. All potentials were measured against the Ag/AgCl reference electrode and were converted to the reversible hydrogen electrode (RHE) potential scale according to $E$ (vs. RHE) = $E$ (vs. Ag/AgCl) + 0.197 V + 0.059 × pH (= 7.4 for $CO_2$-saturated 0.5 M $KHCO_3$).

Gaseous reaction products were quantified using an SRI gas chromatograph (GC) (Multiple Gas Analyzer #3, model 8610C) equipped with a thermal conductivity detector (TCD) and a flame ionization detector (FID). The mole number of gaseous products were calculated from GC peak areas based on standard curves of standard gas samples.

Before measurements, $CO_2$ gas (99.99%) was purged into the cathodic electrolyte at a rate of 10 mL/min (as controlled by a flowmeter) for 30 min and maintained during the $CO_2$RR measurements. During $CO_2$RR, the electrolyte in the cathodic compartment was stirred at 200 rpm. Prior to constant potential measurements, linear sweep voltammetry (LSV) was performed with a scan rate of 10 mV/s from 0 V to –1.8 V vs. Ag/AgCl in $CO_2$-saturated 0.5 M $KHCO_3$. The electrolysis was carried out at –1.1 V, –1.2 V, –1.3 V, and –1.4 V with each run for 40 min (total

experiment run time of 160 min), and the gaseous products were qualitatively and quantitatively analyzed by GC with TCD detection. Liquid products were quantified by 1H NMR spectroscopy at the end of the run.

The faradaic efficiency (FE) of product *x* was calculated according to equation 1:

$$FE_x = \frac{Q_x}{Q_{\text{total}}} = \frac{t z_x F v_x G P}{R T Q_{\text{total}}} = \frac{z_x F mol_x}{Q_{\text{total}}} \quad (1)$$

where $t$ is the sampling time = 1 min; $z_x$ is the number of electrons required to form a molecule of product $x$ ($z_x$ = 2 for CO or $H_2$); $F$ is the Faraday constant = 96485 C/mol; $v_x$ is the volume concentration of product ($x$) measured by GC; $G$ is the flow rate of $CO_2$ = 0.01 L/min; $P$ = 101325 Pa, $R$ = 8.314 J/mol·K; $T$= 298 K; $Q_{\text{total}}$ is the total charge consumed in the electrochemical reaction; and $mol_x$ is the number of moles of product $x$.

The partial current density ($j_x$) of product $x$ was calculated according to equation 2:

$$j_x = \frac{(FE_x) Q_{\text{total}}}{tA} \quad (2)$$

where $FE_x$ is the faradaic efficiency of product $x$; $Q_{\text{total}}$ is the total charge consumed in the electrochemical reaction; $t$ is the sampling time = 1 min; and $A$ is the geometric surface area of the electrode = 1 $cm^2$.

The turnover frequency (*TOF*) of catalysts was calculated according to equation 3:

$$TOF = \frac{mol_x}{t mol_{NC}} \quad (3)$$

where $mol_x$ is the moles of product $x$; $mol_{NC}$ is moles of nanoclusters (catalyst).

**6. Computational methods**

To calculate the number of $CO_2$-accessible metal sites, the whole structure of each NC as determined by crystallography was used directly, avoiding the process of structure relaxation and ligand simplification. A series of conformers for each cluster was generated by taking combinations of phenyl rotations (in 90° steps) and tert-butyl rotations (in 60° steps). These series of conformers were generated using a modified version of the ConfSearch code[s8] wherein the "nangle" variable was adjusted to eliminate symmetrically redundant rotations. For example, when rotating a phenyl ring in 90° intervals, only one 90° rotation was necessary as subsequent rotations give identical structures to either the starting structure or the first rotation due to the 2-fold rotational symmetry of the phenyl group. Therefore, the five conformations generated from [0°,360°] in 90° intervals could be reduced to two conformations. Since the number of conformations scales as $\theta^n$ for $\theta$ rotations across $n$ ligands, we further reduced the number of conformations by separating the ligands into top, middle, and bottom layers. The ligands were divided in half, then one half was assigned as the top layer, the second half as the bottom layer, and the half of the ligands shared between these two layers as the middle layer. Using this scheme,

the number of conformations for a large cluster such as $Au_{44}(C{\equiv}CPh)_{28}$ was reduced from $\theta^{n} = 5^{28}$ to $3(\theta_{symm})^{n/2} = 3(2)^{14} = 49{,}152$, supplying a computationally tractable approximation of the ligand conformations accessible for each cluster. Conformations and surface areas were calculated for each of these three layers.

The accessible surface area of each conformation was then calculated at every atom according to the Shrake-Rupley routine[s9] (Figure S8) implemented in the MDTraj library[s10] using a 1.65 Å probe (the kinetic radius of $CO_2$) and the Golden Section Spiral algorithm to generate a mesh around each atomic center. For each cluster studied herein, the surface area was calculated at an increasing number of mesh points until the difference between iterations became less than 1%. Such convergence was obtained at $10^5$ mesh points, which were used for all surface areas reported herein. Individual conformations were generated in serial, then their surface areas were calculated in parallel through a divide-and-conquer routine across 32 cores to reduce computational time. Using this scheme, the conformations were generated, and their surface areas calculated at ~7 conformations/s; the surface area of each cluster with its various ligand orientations could be screened in ~1 hour. Only metal atoms with a positive surface area, i.e., having a non-zero contact area with $CO_2$, were counted as being accessible. This scheme to binarize the surface areas to "accessible" and "inaccessible" atoms was benchmarked by changing the threshold area—lowest acceptable contact area to deem the atom accessible—from 0 to 2.0 Å$^2$, the results of which are shown in Figure S9. We found that rounding ultrasmall contact areas < 0.1 Å$^2$ to 0 (i.e., inaccessible) gave the most consistent results. The conformation with the greatest number of accessible metals ($N$) was used to represent the surface accessibility for each NC.

## 7. Supplementary Tables

**Table S1.** Crystal data for $\mathbf{Au_{17.67}Ag_{28.33}(C{\equiv}CPh\text{–}\mathit{m}\text{–}F)_{24}Cl_4(PPh_3)_2}$.

| | |
|---|---|
| Empirical formula | $C_{228}H_{126}Au_{17.67}Ag_{28.33}F_{24}C1_4P_2$ |
| Formula weight | 10061.38 |
| Temperature/K | 100 |
| Crystal system | triclinic |
| Space group | *P*-1 |
| *a*/Å | 18.6625(3) |
| *b*/Å | 19.1498(2) |
| *c*/Å | 19.7834(3) |
| *α*/° | 110.0958(12) |
| *β*/° | 116.0798(14) |
| *γ*/° | 96.0693(11) |
| Volume/Å$^3$ | 5680.68(15) |
| *Z* | 1 |
| $\rho_{calc}$ /g cm$^{-3}$ | 2.941 |
| $\mu$/mm$^{-1}$ | 13.859 |
| *F*(000) | 4535 |
| Crystal size/mm$^3$ | 0.086 × 0.080 × 0.030 |
| Radiation | MoK$_\alpha$ (λ = 0.71073 Å) |
| 2$\theta$ range for data collection/° | 3.692 to 50.054 |
| Index ranges | –22 ≤ h ≤ 22, –22 ≤ k ≤ 22, –23 ≤ l ≤ 23 |
| Reflections collected | 103841 |
| Independent reflections | 20068 [$R_{int}$ = 0.0445, $R_{sigma}$ = 0.0390] |
| Data/restraints/parameters | 20068/697/1549 |
| Goodness-of-fit on $F^2$ | 1.013 |
| Final *R* indexes [$I$>=2$\sigma$ ($I$)] | $R_1$ = 0.0308, $wR_2$ = 0.0554 |
| Final *R* indexes [all data] | $R_1$ = 0.0456, $wR_2$ = 0.0588 |
| Largest diff. peak/hole / e Å$^{-3}$ | 1.753/–1.443 |

**Table S2.** Atomic percentages of Au and Ag in the icosahedral $M_{12}$ kernel of $\mathbf{Au_{17.67}Ag_{28.33}(C{\equiv}CPh\text{–}\mathit{m}\text{–}F)_{24}Cl_4(PPh_3)_2}$ determined by X-ray crystallography.

| position | **1** | **2** | **3** | **4** | **5** | **6** |
|---|---|---|---|---|---|---|
| Au | 0.503 | 0.515 | 0.538 | 0.500 | 0.295 | 0.487 |
| Ag | 0.497 | 0.485 | 0.462 | 0.500 | 0.705 | 0.513 |

**Table S3.** Crystal data for **$Au_{19}Ag_{27}(C{\equiv}CPh–\textit{m}–CH_3)_{24}Cl_4(PPh_3)_2(CH_3CN)_4$**.[a]

| | |
|---|---|
| Empirical formula | $C_{260}H_{210}Au_{19}Ag_{27}C1_4P_2N_4$ |
| Formula weight | 10248.90 |
| Temperature/K | 250 |
| Crystal system | triclinic |
| Space group | *P*-1 |
| *a*/Å | 19.1204(2) |
| *b*/Å | 19.4571(2) |
| *c*/Å | 19.7081(2) |
| *α*/° | 106.5328(10) |
| *β*/° | 101.7039(10) |
| *γ*/° | 111.0497(11) |
| Volume/Å$^3$ | 6166.66(13) |
| *Z* | 1 |
| $\rho_{calc}$ /g cm$^{-3}$ | 2.760 |
| $\mu$/mm$^{-1}$ | 13.439 |
| *F*(000) | 4666 |
| Crystal size/mm$^3$ | 0.080 × 0.020 × 0.010 |
| Radiation | MoK$_\alpha$ (λ = 0.71073) |
| 2$\theta$ range for data collection/° | 3.522 to 50.054 |
| Index ranges | $-22 \le h \le 22$, $-23 \le k \le 23$, $-23 \le l \le 23$ |
| Reflections collected | 180531 |
| Independent reflections | 21771 [$R_{int}$ = 0.0875, $R_{sigma}$ = 0.0471] |
| Data/restraints/parameters | 21771/1107/1533 |
| Goodness-of-fit on $F^2$ | 1.004 |
| Final *R* indexes [$I$>=2$\sigma$ ($I$)] | $R_1$ = 0.0277, $wR_2$ = 0.0511 |
| Final *R* indexes [all data] | $R_1$ = 0.0457, $wR_2$ = 0.0552 |
| Largest diff. peak/hole / e Å$^{-3}$ | 1.619/–1.311 |

[a]The crystal structure contains four $CH_3CN$ solvent molecules.

**Table S4.** Atomic percentages of Au or Ag in the icosahedral $M_{12}$ kernel of **$Au_{19}Ag_{27}(C{\equiv}CPh–\textit{m}–CH_3)_{24}Cl_4(PPh_3)_2$** determined by X-ray crystallography.

| position | **1** | **2** | **3** | **4** | **5** | **6** |
|---|---|---|---|---|---|---|
| Au | 0.613 | 0.589 | 0.578 | 0.626 | 0.458 | 0.640 |
| Ag | 0.387 | 0.411 | 0.422 | 0.374 | 0.542 | 0.360 |

**Table S5.** Molecular weights of the alkynyl-protected NCs in g/mol.

| | |
|---|---|
| $Au_nAg_{46-n}(C{\equiv}CPh)_{24}Cl_4(PPh_3)_2$ ($n$ =17) | 9569.99 |
| $Au_{24}Ag_{20}(C{\equiv}CPh^tBu)_{24}Cl_2$ | 10372.62 |
| $Au_{43}(C{\equiv}C^tBu)_{20}$ | 10092.27 |
| $Au_{42}Ag_1(C{\equiv}C^tBu)_{20}$ | 10003.17 |
| $Au_{44}(C{\equiv}CPh)_{28}$ | 11498.03 |
| $Au_{36}(C{\equiv}CPh)_{24}$ | 9517.8 |
| $Au_{23}(C{\equiv}CPh)_{15}$ | 5747.26 |

## 8. Supporting Figures

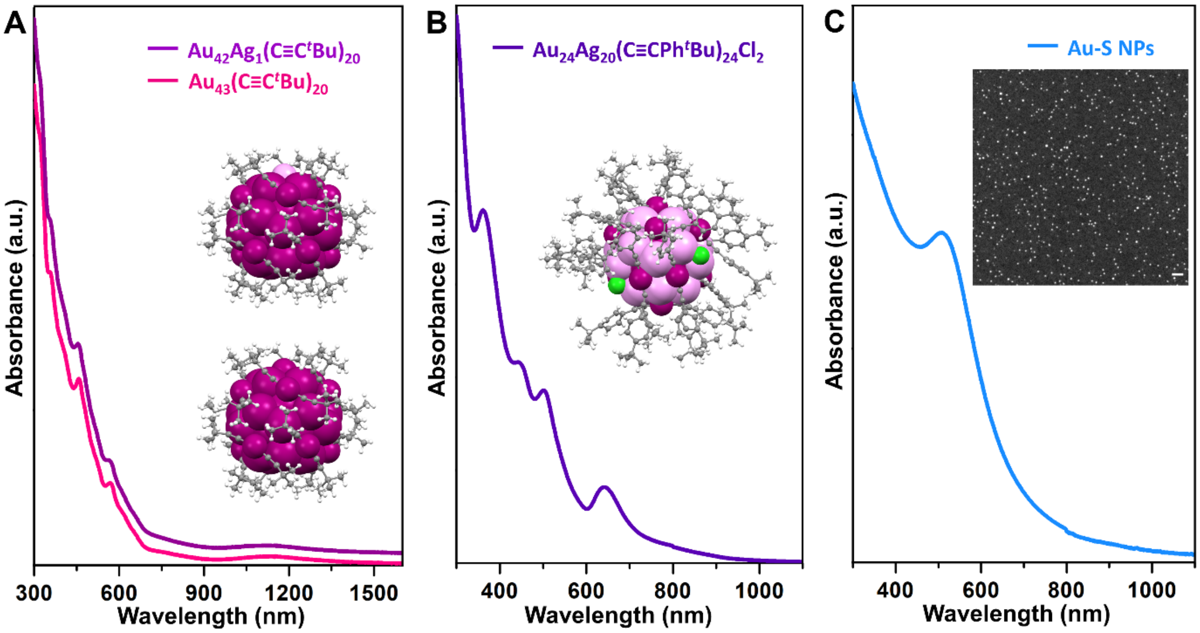


**Figure S1.** UV-vis-NIR absorption spectra of (**A**) $Au_{43}(C{\equiv}C^tBu)_{20}$ and $Au_{42}Ag_1(C{\equiv}C^tBu)_{20}$ NCs, (**B**) $Au_{24}Ag_{20}(C{\equiv}CPh^tBu)_{24}Cl_2$ NCs,[s1] and (**C**) Au–$SC_2H_4Ph$ NPs, inset, scanning transmission electron microscope image of Au–$SC_2H_4Ph$ NPs, scale bar = 20 nm .

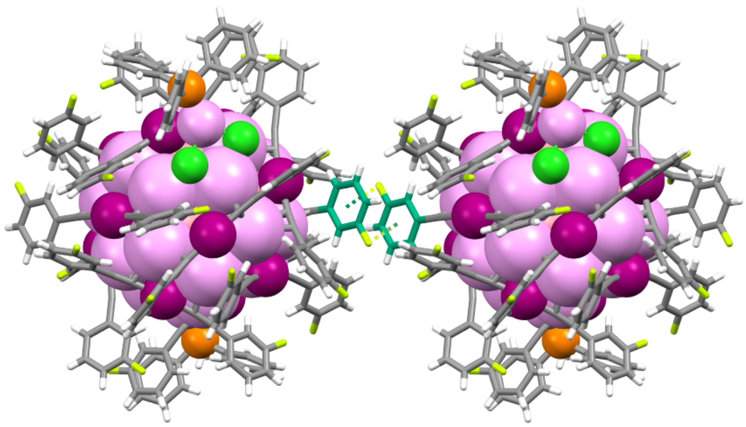

**Figure S2.** Interparticle ligand interactions between two $Au_nAg_{46-n}(C{\equiv}CPh–m–F)_{24}Cl_4(PPh_3)_2$ NCs.

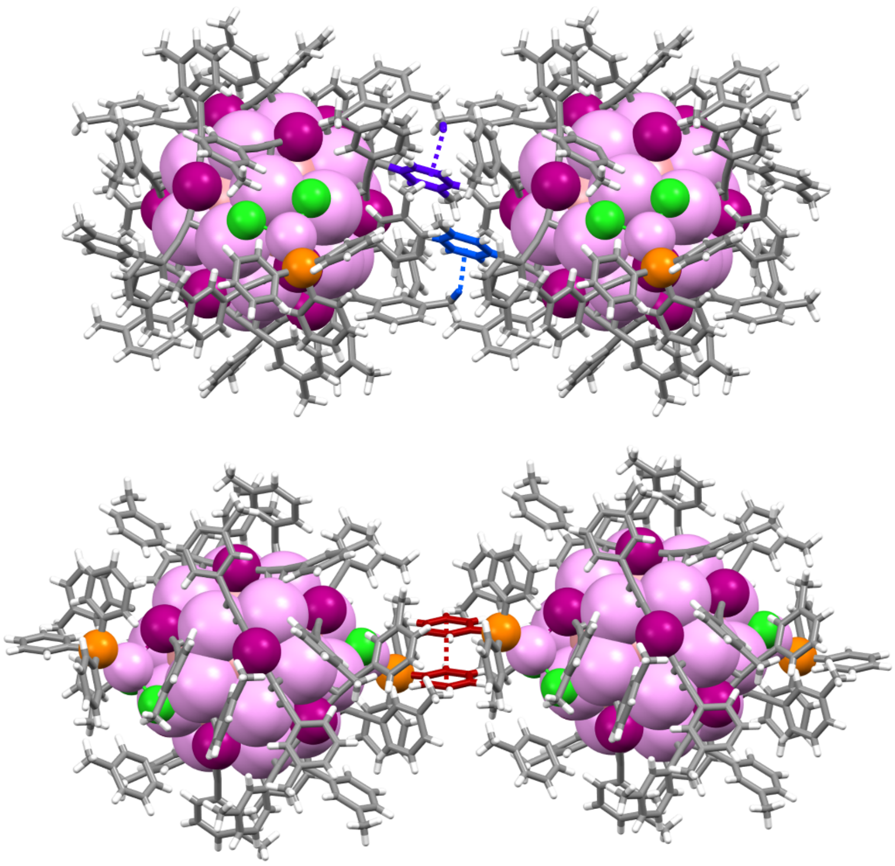

**Figure S3.** Interparticle ligand interactions between two $Au_nAg_{46-n}(C{\equiv}CPh\text{–}m\text{–}CH_3)_{24}Cl_4(PPh_3)_2$ NCs.

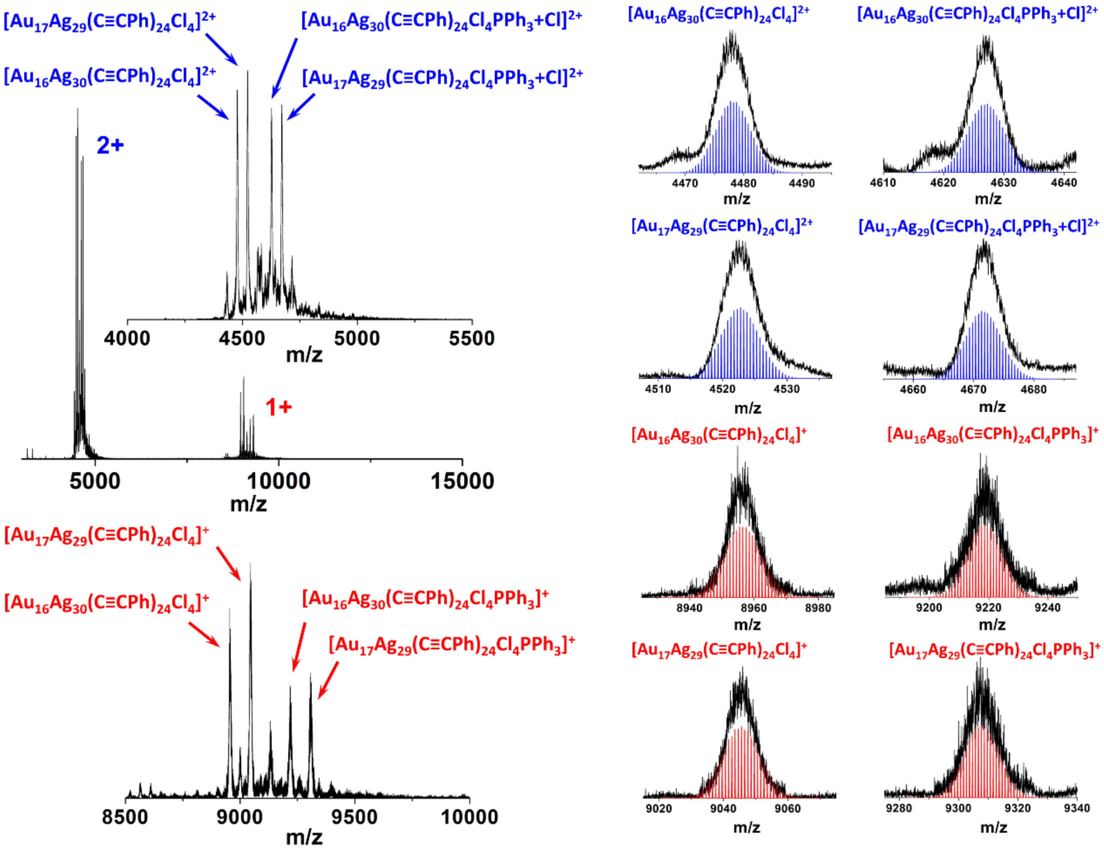


**Figure S4.** ESI-MS spectra of $Au_nAg_{46-n}(C{\equiv}CPh)_{24}Cl_4(PPh_3)_2$, including the isotope peaks corresponding to $[Au_{16}Ag_{30}(C{\equiv}CPh)_{24}Cl_4]^{2+}$, $[Au_{17}Ag_{29}(C{\equiv}CPh)_{24}Cl_4]^{2+}$, $[Au_{16}Ag_{30}(C{\equiv}CPh)_{24}Cl_4PPh_3{+}Cl]^{2+}$, $[Au_{17}Ag_{29}(C{\equiv}CPh)_{24}Cl_4PPh_3{+}Cl]^{2+}$ (calculated isotope peaks are shown as blue lines); and $[Au_{16}Ag_{30}(C{\equiv}CPh)_{24}Cl_4]^{+}$, $[Au_{17}Ag_{29}(C{\equiv}CPh)_{24}Cl_4]^{+}$, $[Au_{16}Ag_{30}(C{\equiv}CPh)_{24}Cl_4PPh_3]^{+}$, $[Au_{17}Ag_{29}(C{\equiv}CPh)_{24}Cl_4PPh_3]^{+}$ (calculated isotope peaks are shown as red lines). The $Au_nAg_{46-n}(C{\equiv}CPh)_{24}Cl_4(PPh_3)_2$ NCs lose one or two $PPh_3$ ligands during the ionization.

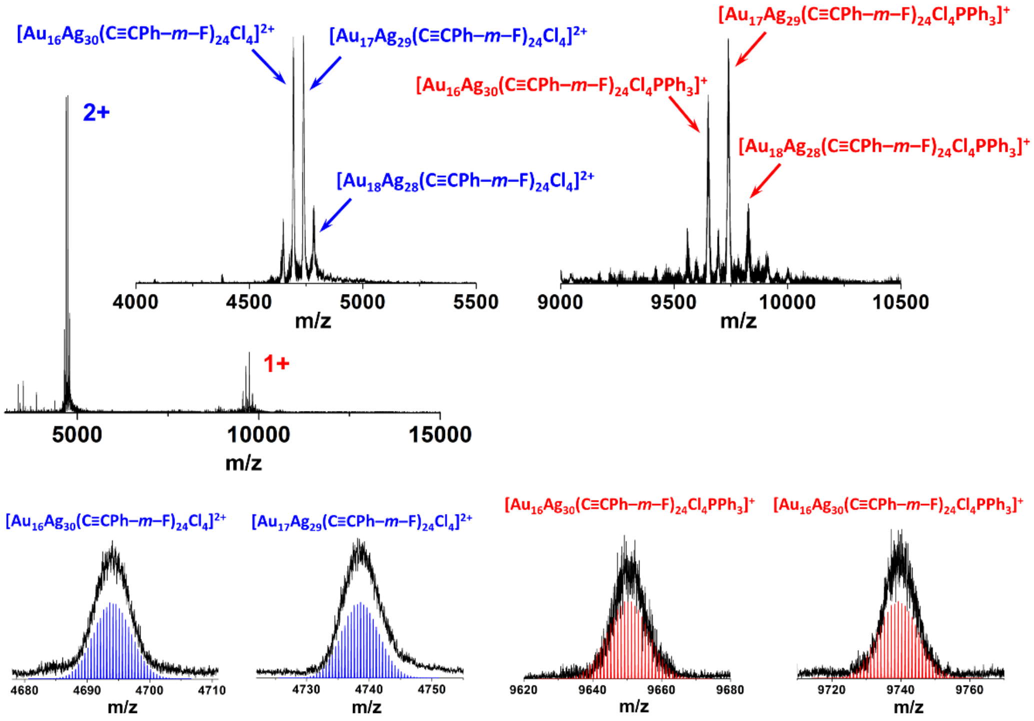


**Figure S5.** ESI-MS spectra of $Au_nAg_{46-n}(C{\equiv}CPh\text{–}m\text{–}F)_{24}Cl_4(PPh_3)_2$, including the isotope peaks corresponding to $[Au_{16}Ag_{30}(C{\equiv}CPh\text{–}m\text{–}F)_{24}Cl_4]^{2+}$, $[Au_{17}Ag_{29}(C{\equiv}CPh\text{–}m\text{–}F)_{24}Cl_4]^{2+}$ (calculated isotope peaks are shown as blue lines); and $[Au_{16}Ag_{30}(C{\equiv}CPh\text{–}m\text{–}F)_{24}Cl_4PPh_3]^{+}$, $[Au_{17}Ag_{29}(C{\equiv}CPh\text{–}m\text{–}F)_{24}Cl_4PPh_3]^{+}$ (calculated isotope peaks are shown as red lines). The $Au_nAg_{46-n}(C{\equiv}CPh\text{–}m\text{–}F)_{24}Cl_4(PPh_3)_2$ NCs lose one or two $PPh_3$ ligands during the ionization.

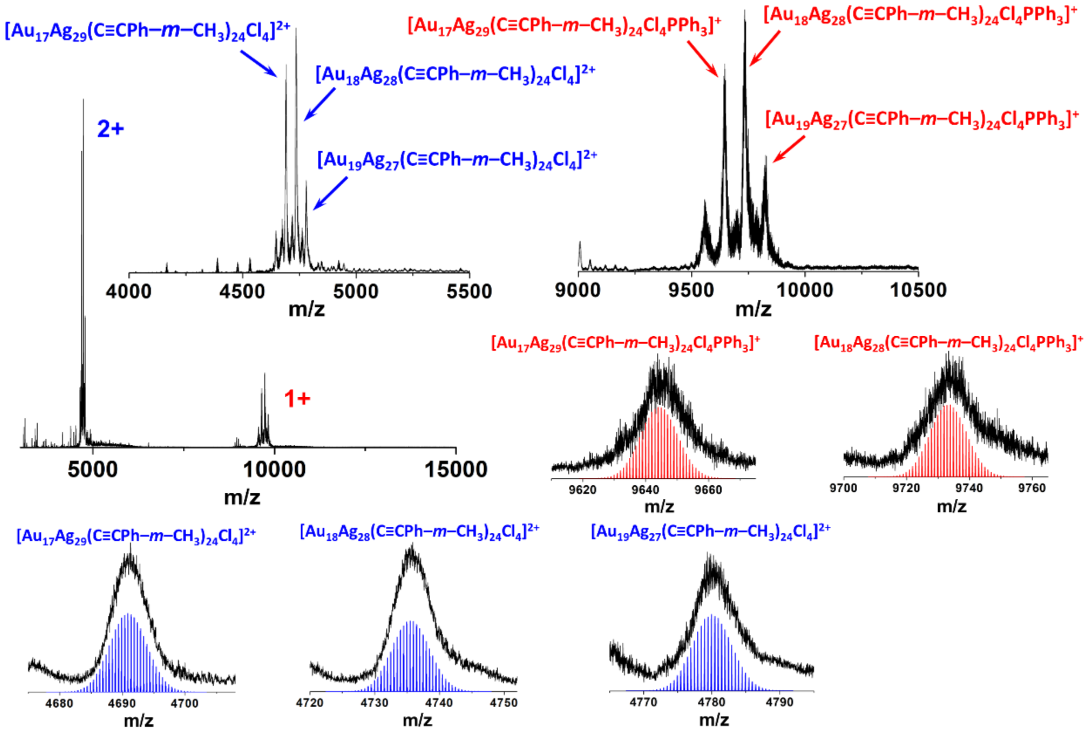


**Figure S6.** ESI-MS spectra of $Au_nAg_{46-n}(C{\equiv}CPh{-}m{-}CH_3)_{24}Cl_4(PPh_3)_2$, including the isotope peaks corresponding to $[Au_{17}Ag_{29}(C{\equiv}CPh{-}m{-}CH_3)_{24}Cl_4]^{2+}$, $[Au_{18}Ag_{28}(C{\equiv}CPh{-}m{-}CH_3)_{24}Cl_4]^{2+}$, $[Au_{19}Ag_{27}(C{\equiv}CPh{-}m{-}CH_3)_{24}Cl_4]^{2+}$ (calculated isotope peaks are shown as blue lines); and $[Au_{17}Ag_{29}(C{\equiv}CPh{-}m{-}CH_3)_{24}Cl_4PPh_3]^{+}$, $[Au_{18}Ag_{28}(C{\equiv}CPh{-}m{-}CH_3)_{24}Cl_4PPh_3]^{+}$, $[Au_{18}Ag_{28}(C{\equiv}CPh{-}m{-}CH_3)_{24}Cl_4PPh_3]^{+}$ (calculated isotope peaks are shown as red lines). The $Au_nAg_{46-n}(C{\equiv}CPh{-}m{-}CH_3)_{24}Cl_4(PPh_3)_2$ NCs lose one or two $PPh_3$ ligands during the ionization.

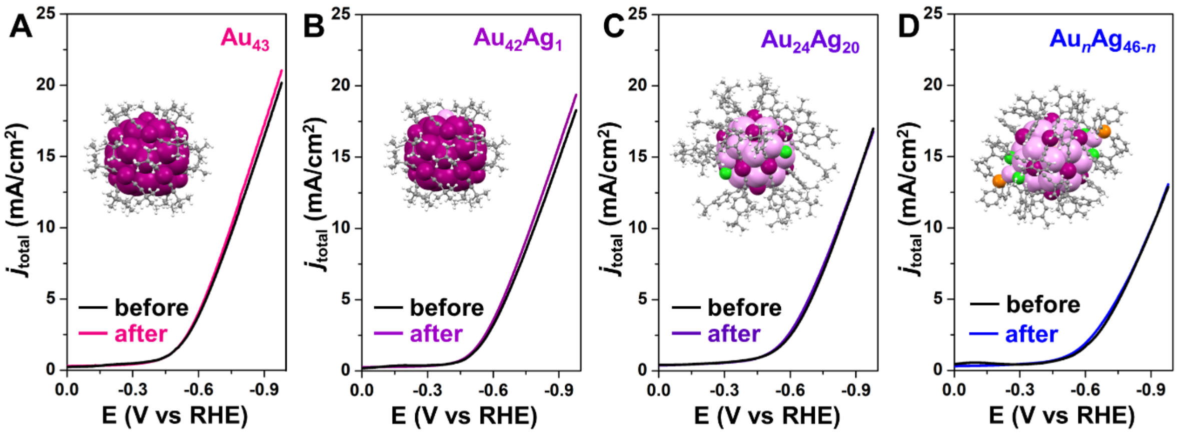


**Figure S7.** LSV before and after chronoamperometric $CO_2RR$ catalysis for (A) $Au_{43}(C{\equiv}C^{t}Bu)_{20}$, (B) $Au_{42}Ag_{1}(C{\equiv}C^{t}Bu)_{20}$, (C) $Au_{24}Ag_{20}(C{\equiv}CPh^{t}Bu)_{24}Cl_{2}$, and (D) $Au_{n}Ag_{46-n}(C{\equiv}CR)_{24}Cl_{4}(PPh_{3})_{2}$.

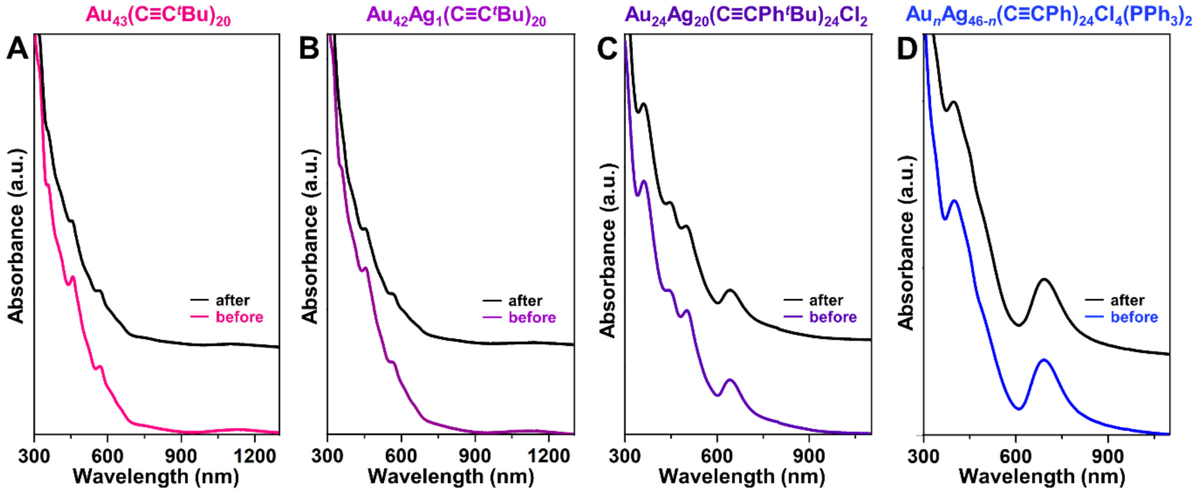


**Figure S8.** UV-vis-NIR absorption spectra of (**A**) $Au_{43}(C{\equiv}C^{t}Bu)_{20}$, (**B**) $Au_{42}Ag_{1}(C{\equiv}C^{t}Bu)_{20}$, (**C**) $Au_{24}Ag_{20}(C{\equiv}CPh^{t}Bu)_{24}Cl_{2}$, and (**D**) $Au_{n}Ag_{46-n}(C{\equiv}CPh)_{24}Cl_{4}(PPh_{3})_{2}$ NCs before (colored lines) and after (black lines) electrolysis. The black curves are offset vertically for clarity.

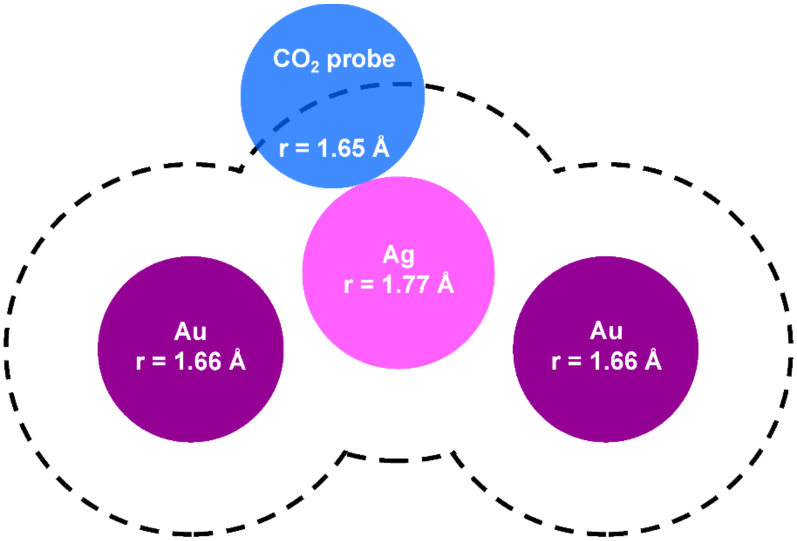


**Figure S9**. Illustration of accessible surface area calculation by the Shrake-Rupley method using the kinetic radius of $CO_2$ (1.65 Å) as a probe.

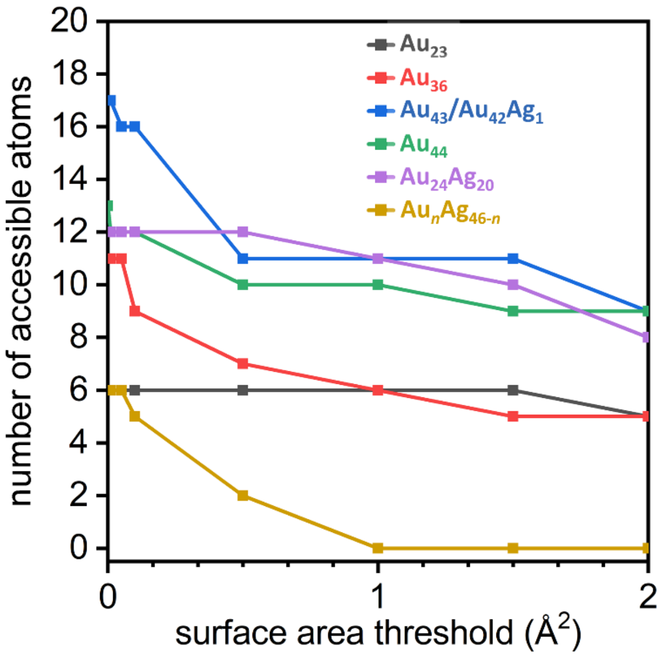


**Figure S10.** Plot of the number of accessible metal sites of different NCs for different threshold surface areas (black represents $Au_{23}(C{\equiv}C^{t}Bu)_{15}$; red represents $Au_{36}(C{\equiv}CPh)_{24}$; blue represents $Au_{43}(C{\equiv}C^{t}Bu)_{20}/Au_{42}Ag_{1}(C{\equiv}C^{t}Bu)_{20}$, green represents $Au_{44}(C{\equiv}CPh)_{28}$, violet represents $Au_{24}Ag_{20}(C{\equiv}CPh^{t}Bu)_{24}Cl_{2}$, and yellow represents $Au_{n}Ag_{46-n}(C{\equiv}CPh)_{24}Cl_{4}(PPh_{3})_{2}$.

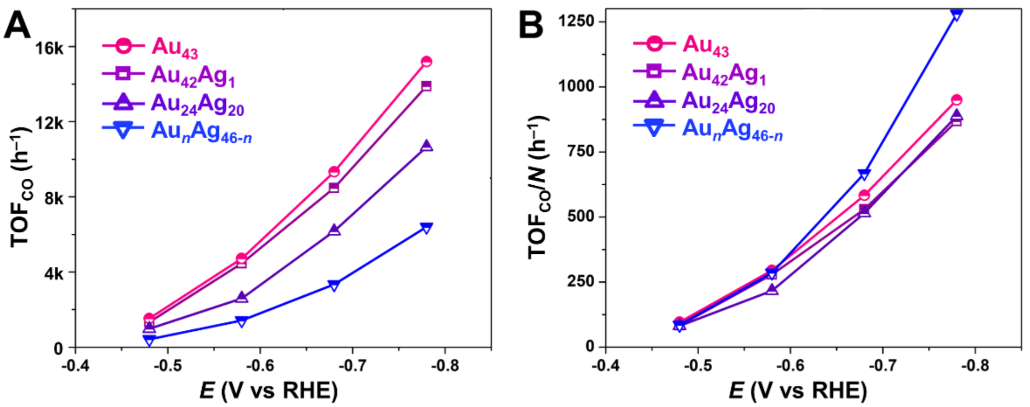


**Figure S11.** Potential-dependent (**A**) $TOF_{CO}$ and (**B**) $TOF_{CO}/N$ of NC-based catalysts. NCs include $Au_{43}(C{\equiv}C^tBu)_{20}$ (magenta), $Au_{42}Ag_1(C{\equiv}C^tBu)_{20}$ (purple), $Au_{24}Ag_{20}(C{\equiv}CPh^tBu)_{24}Cl_2$ (indigo), and $Au_nAg_{46-n}(C{\equiv}CPh)_{24}Cl_4(PPh_3)_2$ (blue). $N$ corresponds to the number of accessible metal sites for each NC.

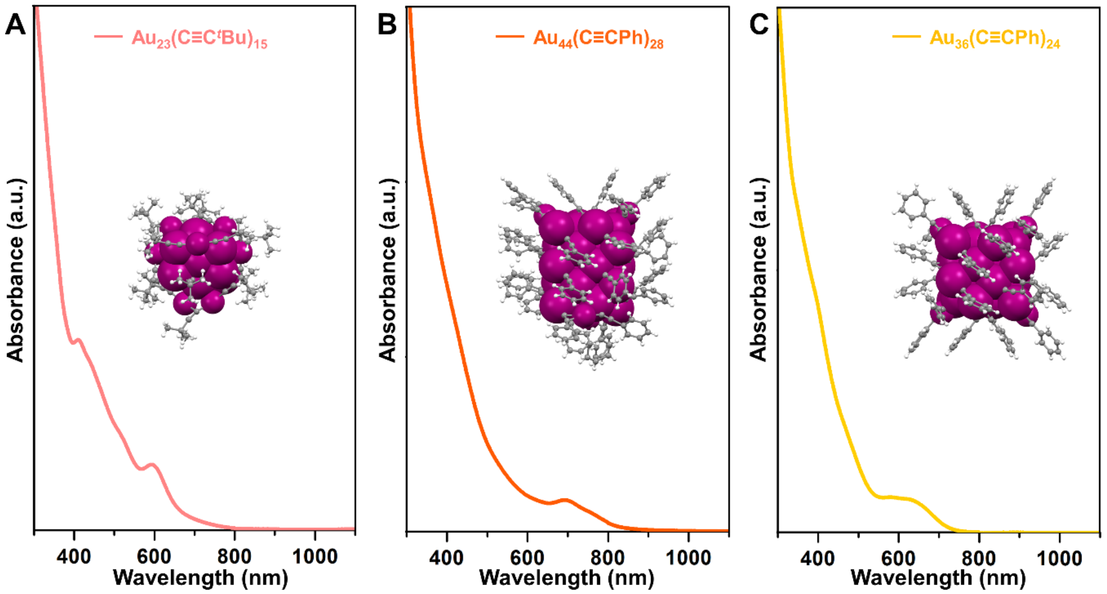


**Figure S12.** UV-vis-NIR absorption spectra for (**A**) $Au_{23}(C{\equiv}C^tBu)_{15}$, (**B**) $Au_{44}(C{\equiv}CPh)_{28}$, and (**C**) $Au_{36}(C{\equiv}CPh)_{24}$ NCs.

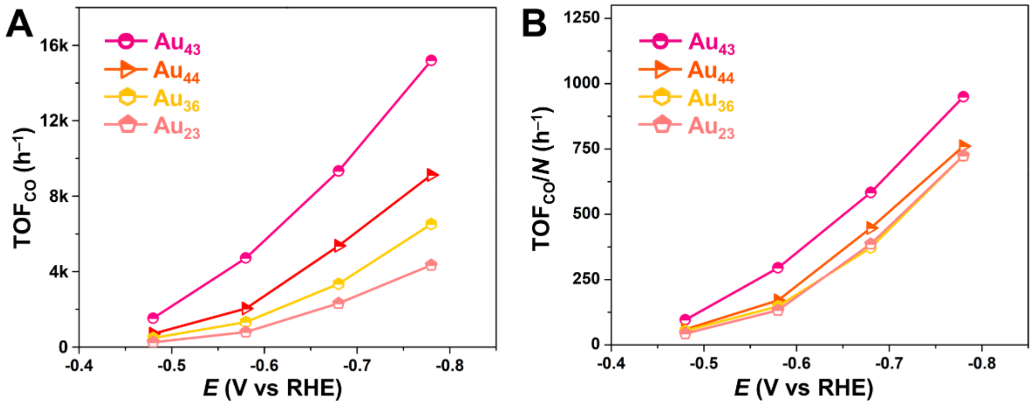


**Figure S13.** Potential-dependent $TOF_{CO}$ and $TOF_{CO}/N$ of NC-based catalysts. NCs include $Au_{43}(C{\equiv}C^tBu)_{20}$ (magenta), $Au_{23}(C{\equiv}C^tBu)_{15}$ (pink), $Au_{44}(C{\equiv}CPh)_{28}$ (orange), and $Au_{36}(C{\equiv}CPh)_{24}$ (yellow). $N$ corresponds to the number of accessible metal sites for each NC.

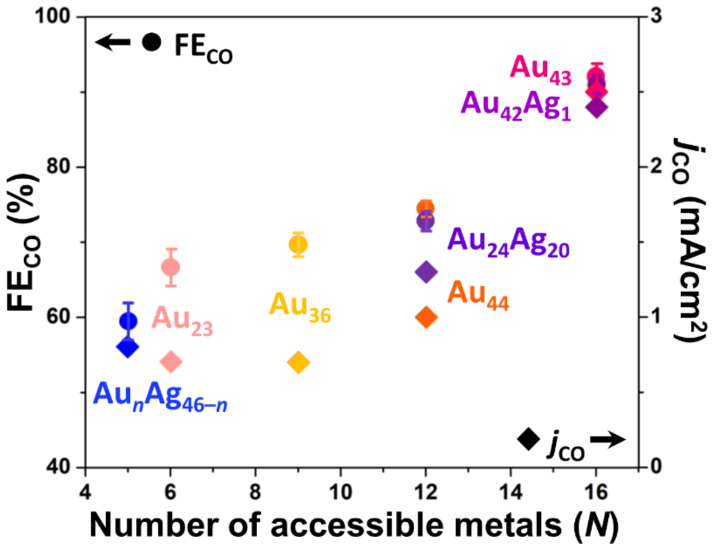


**Figure S14.** The relationship between the number of accessible metal sites and $FE_{CO}$ and $j_{CO}$.

## 9. Supporting References


(s1) Wen, F.; Englert, U.; Gutrath, B. S.; Simon, U. Crystal Structure, Electrochemical and Optical Properties of $[Au_9(PPh_3)_8](NO_3)_3$. *Eur. J. Inorg. Chem.* **2008**, 106–111.

(s2) Xu, J.; Xiong, L.; Cai, X.; Tang, S.; Tang, A.; Liu, X.; Pei, Y.; Zhu, Y. Evolution from Superatomic $Au_{24}Ag_{20}$ Monomers into Molecular-like $Au_{43}Ag_{38}$ Dimeric Nanoclusters. *Chem. Sci.* **2022**, *13*, 2778–2782.

(s3) Tang, Y.; Sun, F.; Ma, X.; Qin, L.; Ma, G.; Tang, Q.; Tang, Z. Alkynyl and Halogen Co-Protected $(AuAg)_{44}$ Nanoclusters: A Comparative Study on Their Optical Absorbance, Structure, and Hydrogen Evolution Performance. *Dalton Trans.* **2022**, *51*, 7845–7850.

(s4) Wan, X.-K.; Guan, Z.-J.; Wang, Q.-M. Homoleptic Alkynyl-Protected Gold Nanoclusters: $Au_{44}(PhC{\equiv}C)_{28}$ and $Au_{36}(PhC{\equiv}C)_{24}$. *Angew. Chem. Int. Ed.* **2017**, *56*, 11494–11497.

(s5) Dolomanov, O. V.; Bourhis, L. J.; Gildea, R. J.; Howard, J. A.; Puschmann, H. OLEX2: A Complete Structure Solution, Refinement and Analysis Program. *J. Appl. Cryst.* **2009**, *42*, 339–341.

(s6) Sheldrick, G. M. SHELXT – Integrated Space-Group and Crystal-Structure Determination. *Acta. Cryst.* **2015**, *A71*, 3–8.

(s7) Sheldrick, G. M. Crystal Structure Refinement with SHELXL. *Acta. Cryst.* **2015**, *C71*, 3–8.

(s8) Meana-Pañeda, R.; Truhlar, D. G. ConfSearch: A Conformational Search Program. *ConfSearch: A conformational search program, University of Minnesota, Minneapolis*. Version 2015. Department of Chemistry and Supercomputing Institute, University of Minnesota, Minneapolis 2015.

(s9) Shrake, A.; Rupley, J. A. Environment and Exposure to Solvent of Protein Atoms. Lysozyme and Insulin. *J. Mol. Biol.* **1973**, *79*, 351–371.

(s10) McGibbon, R. T.; Beauchamp, K. A.; Harrigan, M. P.; Klein, C.; Swails, J. M.; Hernández, C. X.; Schwantes, C. R.; Wang, L.-P.; Lane, T. J.; Pande, V. S. MDTraj: A Modern Open Library for the Analysis of Molecular Dynamics Trajectories. *Biophys. J.* **2015**, *109*, 1528−1532.
